# Supplementary material for: A fault-intrusion-tolerant system and deadline-aware algorithm for scheduling scientific workflow in the cloud
Source: PeerJ Comput Sci. 2021 Nov 2;7:e747. doi: 10.7717/peerj-cs.747 (PMC8576554; doi:10.7717/peerj-cs.747)
Supplement: Supplemental Information 6 [file peerj-cs-07-747-s006.zip › Myproject1.1/docs/org/cloudbus/cloudsim/UtilizationModelPlanetLabInMemory.html]

UtilizationModelPlanetLabInMemory


JavaScript is disabled on your browser.


- Overview
- Package
- Class
- Use
- Tree
- Deprecated
- Index
- Help

- Prev Class
- Next Class

- Frames
- No Frames

- All Classes

- Summary:
- Nested |
- Field |
- Constr |
- Method

- Detail:
- Field |
- Constr |
- Method


org.cloudbus.cloudsim

## Class UtilizationModelPlanetLabInMemory

- java.lang.Object
- - org.cloudbus.cloudsim.UtilizationModelPlanetLabInMemory

- All Implemented Interfaces:
  :   UtilizationModel

  ---

    

  ```
  public class UtilizationModelPlanetLabInMemory
  extends java.lang.Object
  implements UtilizationModel
  ```

  The Class UtilizationModelPlanetLab.

- - ### Constructor Summary

    Constructors

    Constructor and Description

    `UtilizationModelPlanetLabInMemory(java.lang.String inputPath,
    double schedulingInterval)`

    Instantiates a new utilization model PlanetLab.

    `UtilizationModelPlanetLabInMemory(java.lang.String inputPath,
    double schedulingInterval,
    int dataSamples)`

    Instantiates a new utilization model PlanetLab with variable data samples.
  - ### Method Summary

    Methods

    | Modifier and Type | Method and Description |
    | --- | --- |
    | `double` | `getSchedulingInterval()` Gets the scheduling interval. |
    | `double` | `getUtilization(double time)` Returns utilization in percents according to the time. |
    | `void` | `setSchedulingInterval(double schedulingInterval)` Sets the scheduling interval. |

    - ### Methods inherited from class java.lang.Object

      `clone, equals, finalize, getClass, hashCode, notify, notifyAll, toString, wait, wait, wait`

- - ### Constructor Detail


    - #### UtilizationModelPlanetLabInMemory

      ```
      public UtilizationModelPlanetLabInMemory(java.lang.String inputPath,
                                       double schedulingInterval)
                                        throws java.lang.NumberFormatException,
                                               java.io.IOException
      ```

      Instantiates a new utilization model PlanetLab.

      Parameters:
      :   `inputPath` - the input path

      Throws:
      :   `java.lang.NumberFormatException` - the number format exception
      :   `java.io.IOException` - Signals that an I/O exception has occurred.


    - #### UtilizationModelPlanetLabInMemory

      ```
      public UtilizationModelPlanetLabInMemory(java.lang.String inputPath,
                                       double schedulingInterval,
                                       int dataSamples)
                                        throws java.lang.NumberFormatException,
                                               java.io.IOException
      ```

      Instantiates a new utilization model PlanetLab with variable data samples.

      Parameters:
      :   `inputPath` - the input path
      :   `dataSamples` - number of samples in the file

      Throws:
      :   `java.lang.NumberFormatException` - the number format exception
      :   `java.io.IOException` - Signals that an I/O exception has occurred.
  - ### Method Detail


    - #### getUtilization

      ```
      public double getUtilization(double time)
      ```

      **Description copied from interface: `UtilizationModel`**

      Returns utilization in percents according to the time.

      **Specified by:**
      :   `getUtilization` in interface `UtilizationModel`

      Parameters:
      :   `time` - the time

      Returns:
      :   utilization percentage


    - #### setSchedulingInterval

      ```
      public void setSchedulingInterval(double schedulingInterval)
      ```

      Sets the scheduling interval.

      Parameters:
      :   `schedulingInterval` - the new scheduling interval


    - #### getSchedulingInterval

      ```
      public double getSchedulingInterval()
      ```

      Gets the scheduling interval.

      Returns:
      :   the scheduling interval


- Overview
- Package
- Class
- Use
- Tree
- Deprecated
- Index
- Help

- Prev Class
- Next Class

- Frames
- No Frames

- All Classes

- Summary:
- Nested |
- Field |
- Constr |
- Method

- Detail:
- Field |
- Constr |
- Method
